# Hepatitis C Virus Infection Is Associated With an Increased Risk of Active Tuberculosis Disease: A Nationwide Population-Based Study

**DOI:** 10.1097/MD.0000000000001328

**Published:** 2015-08-21

**Authors:** Ping-Hsun Wu, Yi-Ting Lin, Kun-Pin Hsieh, Hung-Yi Chuang, Chau-Chyun Sheu

**Affiliations:** From the Division of Nephrology (P-HW), Department of Internal Medicine; Department of Family Medicine (Y-TL); Division of Pulmonary and Critical Care Medicine (C-CS), Department of Internal Medicine; Department of Community Medicine (H-YC); Department of Public Health (Y-TL, H-YC), College of Life Science; School of Pharmacy (K-PH), College of Pharmacy; Graduate Institute of Medicine (P-HW); and Department of Internal Medicine (C-CS), College of Medicine, Kaohsiung Medical University, Kaohsiung, Taiwan.

## Abstract

Tuberculosis (TB) and hepatitis C virus (HCV) infection contribute to major disease mortality and morbidity worldwide. However, the causal link between HCV infection and TB risk remains unclear. We conducted a population-based cohort study to elucidate the association between HCV infection and TB disease by analyzing Taiwan National Health Insurance Database. We enrolled 5454 persons with HCV infection and 54,274 age- and sex-matched non-HCV-infected persons between January 1998 and December 2007. Time-dependent Cox proportional hazards regression analysis was used to measure the association between HCV infection and active TB disease. Incidence rate of active TB disease was higher among HCV infection than in control (134.1 vs 89.1 per 100,000 person-years; incidence rate ratio 1.51; *P* = 0.014). HCV infection was significantly associated with active TB disease in multivariate Cox regression (adjusted hazard ratio [HR] 3.20; 95% confidence interval [CI], 1.85–5.53; *P* < 0.001) and competing death risk event analysis (adjusted HR 2.11; 95% CI, 1.39–3.20; *P* < 0.001). Multivariate stratified analysis further revealed that HCV infection was a risk of active TB disease in most strata. This nationwide cohort study suggests that HCV infection is associated with a higher risk of developing active TB disease.

## Abstract 2

Supplemental Digital Content is available in the text

## INTRODUCTION

Tuberculosis (TB), an infectious disease caused by *Mycobacterium tuberculosis*, is the most prevalent infectious disease and the major leading cause of death worldwide, especially in developing countries. According to the Global Tuberculosis Report by World Health Organization, there were an estimated 8.6 million incident cases of TB and approximately 1.3 million people died of TB in 2012.^1^ It is endemic in south-eastern Asia, as well as Taiwan. An epidemiological study declared the incidence of active TB disease in Taiwan was 74 per 100,000 person-years.^2^

TB is considered as an immunodeficiency-related infection. Our recent work demonstrated that liver cirrhosis was associated with increased risk of active TB disease.^3^ Liver cirrhosis, and subsequent complications of both hepatitis B virus (HBV) and hepatitis C virus (HCV) infections are major health problems in Taiwan.^4^ However, a comprehensive evaluation from HCV patients without cirrhosis remains unavailable. HCV infection is one of the contributing factors for developing TB infection is our hypothesis. Previous observation study showed that HCV infection and TB share the same high risks population, especially in homeless people,^5^ prisoners,^6^ and human immunodeficiency virus (HIV) patients.^7^ Furthermore, one case-control study using the US Veterans data demonstrated that HCV infection is associated with TB disease.^8^ However, results from this hospital-based, case-control study cannot be extended to the general population. In order to fill this knowledge gap, this nationwide cohort study analyzed healthcare data to clarify the association between HCV and active TB disease using large-scale data from the Taiwan National Health Insurance (NHI).

## METHODS

### Study Design and Data Source

Data analyzed in this study were retrieved from the Taiwan National Health Insurance Research Database (NHIRD). Taiwan launched a compulsory social insurance program, the NHI program, to provide health care for all residents beginning in 1995. The Taiwan NHIRD includes complete outpatient visits, hospital admissions, prescriptions, disease, and vital status for 99% of the 23 million population of Taiwan. We established the longitudinal medical history of each beneficiary by linking several computerized administrative claims datasets. This study was approved by the Institutional Review Board of Kaohsiung Medical University Hospital (KMUH-IRB-EXEMPT-20130060).

### Study Population and Definition of HCV Infection

**FIGURE 1:**
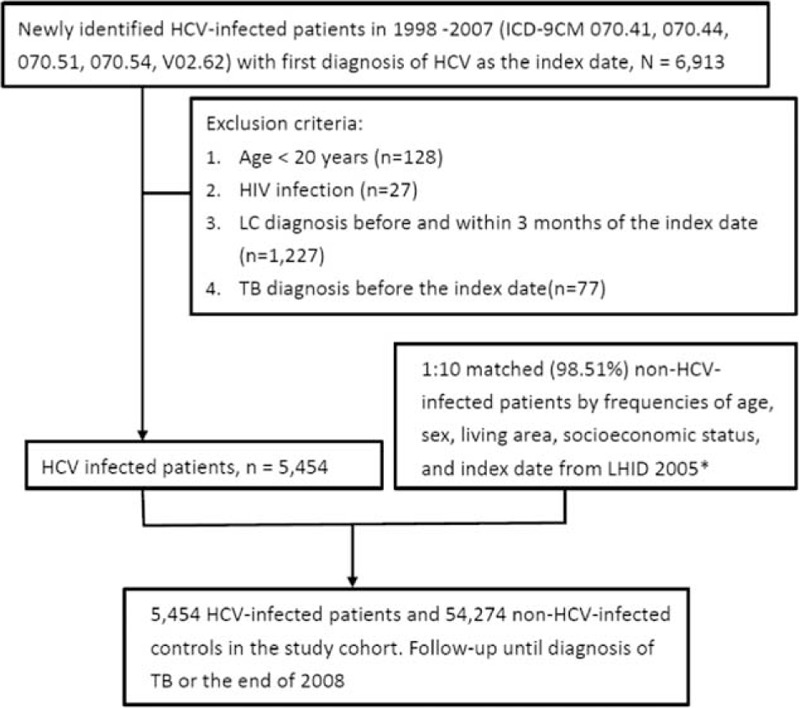
Schematic diagram. HCV = hepatitis C virus, HIV = human immunodeficiency virus, ICD-9-CM = International Classification of Diseases, 9th Revision, Clinical Modification, LC = liver cirrhosis, LHID = Longitudinal Health Insurance Database, TB = tuberculosis. ^∗^Selected normal controls met the same exclusion criteria from the HCV cohort.

The definition of HCV infection required the International Classification of Disease, 9th Revision, Clinical Modification (ICD-9) codes 070.41, 070.44, 070.51, 070.54, 070.7, and V02.62. Patients considered HCV infection by one hospitalization diagnosis or >2 ambulatory visits for a principal diagnosis from 1998 until the end of 2007. The index date for patients with HCV infection was the date of their first medical visit with ICD-9-CM codes for HCV. Individuals <20 years and those with HIV infection (ICD-9 code 042) (n = 27) or liver cirrhosis (ICD-9 codes 571.2, 571.5, 571.6) were excluded before and within 3 months of index date (n = 1227). Patients with TB disease before the index date (n = 77) were also excluded. The control cohort was extracted from 1,000,000 randomly selected people of the NHIRD. We excluded the subjects with diagnoses of HCV from 1998 to 2007, and subjects with diagnoses of active TB disease from 1996 to the index date. We controlled the potential confounding by age, sex, and calendar year through an individual matching technique. The matched controls met the same exclusion criteria from the HCV cohort. For each HCV case, we selected 10 controls with the same age, sex, geographic area, socioeconomic status, and index date as the HCV case by using simple random sampling method (Figure 1). The income served as a proxy indicator of economic status, which was classified as 1 of 3 categories: fixed premium and dependent, New Taiwan Dollars (NTD) <20,000 monthly, or NTD ≥20,000 monthly (US$1 = NTD32.1 in 2008).

### Definition of TB and Comorbidities

Participants were classified as having incident active TB disease by diagnosis of TB with at least 2 ambulatory visits or hospitalizations (ICD-9 codes 010–018). The diagnosis of TB was further confirmed by claiming for 2 or more types of anti-TB drugs for >60 days within 180 days. Rifampin, isoniazid, pyrazinamide, ethambutol, aminogylcoside, prothionamide, cycloserine, and quinolone were defined as anti-TB drugs. Comorbidities in this study, including HBV infection (ICD-9 code 070.2, 070.3, and V02.61), cancers (ICD-9 codes 140–208), diabetic mellitus (DM) (ICD-9 code 250), chronic obstructive pulmonary disease (COPD) (ICD-9 codes 491, 492, and 496), silicosis (ICD-9 codes 501–504), chronic kidney disease (CKD) (ICD-9 code 585), autoimmune diseases (ICD-9 codes 710 and 714), drug abuse (cocaine, amphetamine or related acting sympathomimetic agents, and heroin) (ICD-9 codes 304.2–304.4, 305.7, and 965.01), and organ transplantation (ICD-9 codes 996 and V042), were recognized based on >2 ambulatory visits or hospitalization once. We also used ICD-9 codes to define alcoholism. The corresponding ICD-9 codes for diseases examined in this study were summarized in the Supplementary Table, http://links.lww.com/MD/A374. In addition, we retrieved immunosuppressive agents, which included chemotherapy drugs, steroid, and biological agents during observation period. The immunosuppressive agent users were defined as medications used >28 days during observation period. The endpoint was defined as the date of first diagnosis of TB, death, or the end of the year of 2008.

### Statistical Analysis

Normally distributed continuous data were expressed as means ± standard deviations. Parametric continuous data were compared by Student *t* test, and categorical data were compared by χ^2^ test. The differences between proportions of individuals that developed active TB disease in the HCV-infected and non-HCV-infected control groups were analyzed by the Kaplan–Meier method. We also tested the Cox proportional hazard regression and a log-rank test. The proportional hazard assumption in the Cox model was tested using Schoenfeld residuals trend tests, which examined the interactions between predictors and event time. Interactions between predictors and event time were noted, so those that failed the assumption or were deemed to be time dependent were entered as continuous time-dependent covariates. Therefore, hazard ratios (HRs) for variables treated as time-dependent covariates varied over time. In addition, time-dependent covariate was also applied in multivariate Cox proportional hazard model with stepwise elimination to analyze independent risk factors for active TB disease. Furthermore, death before the development of active TB disease was considered competing event. We modified Cox proportional hazards models in the presence of competing risk event after adjusting for age, sex, and underlying comorbidities.^9^ The association between HCV infection and active TB disease was further analyzed in different strata by age, sex, and comorbidities. A 2-sided *P* <0.05 was considered statistically significant. Analyses were performed using the SAS statistical package version 9.2 (SAS Institute, Cary, NC).

## RESULTS

### Study Population

The study population consists of 5454 HCV-infected patients (cases), with the mean age of 51.8 ± 15.3 years, and 54,274 matched, non-HCV-infected patients (controls), with the mean age of 51.7 ± 15.3 years. Baseline characteristics and selected comorbid medical disorders are demonstrated in Table 1. HCV-infected patients had more comorbidities than controls, including alcoholism, HBV infection, DM, CKD, autoimmune disease, COPD, cancer, organ transplantation, and drug abuse. HCV-infected patients also had more immunosuppressive agents (chemotherapy drugs, steroid, and biological agents) used than controls.

**TABLE 1:**
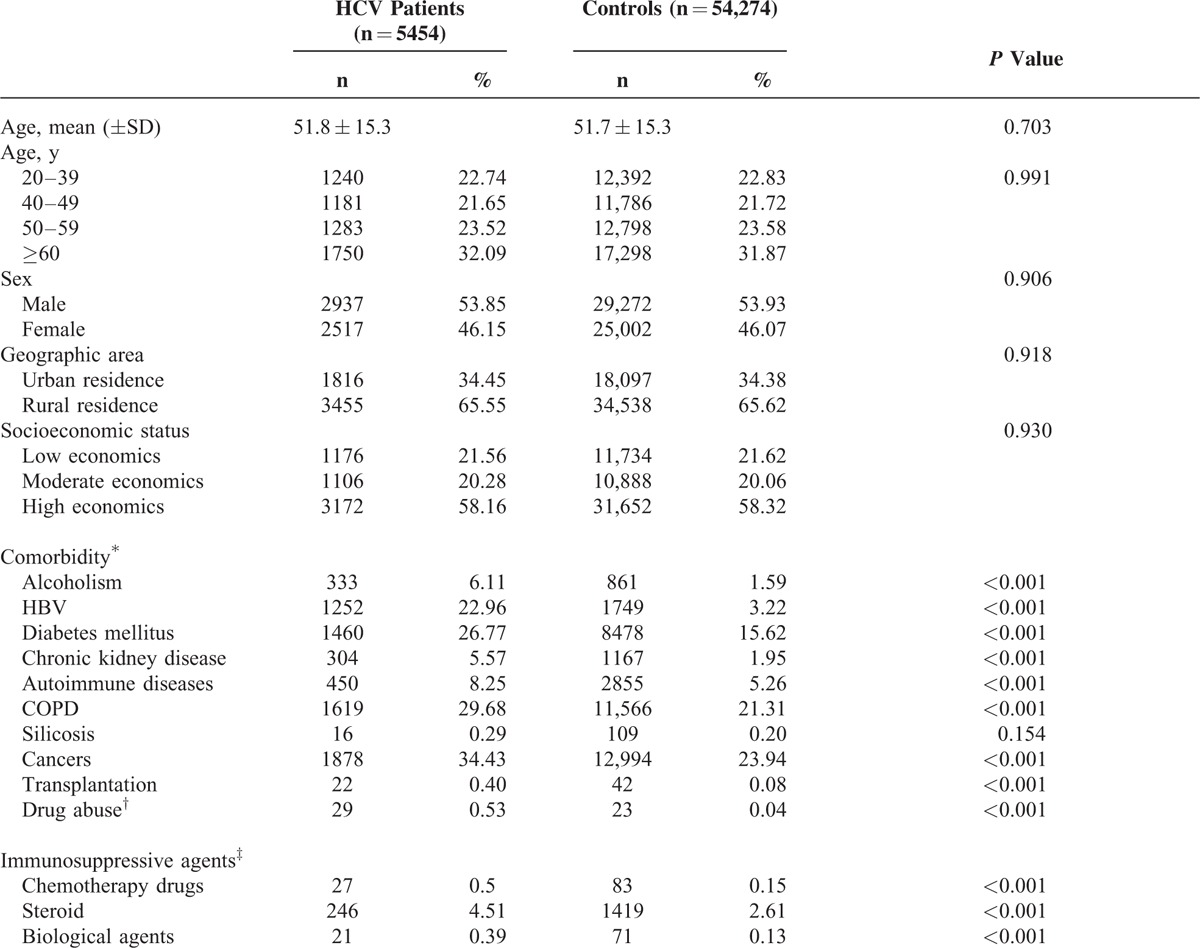
Baseline Characteristics Between HCV-Infected Patients and Non-HCV-Infected Controls

### Risk of Developing Active TB Disease Between HCV Patients and Controls

**FIGURE 2:**
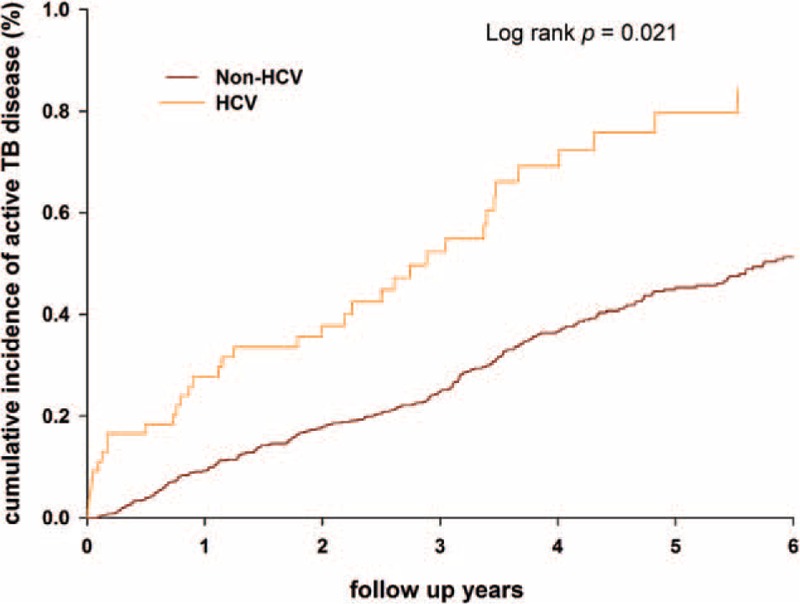
Cumulative incidences of active tuberculosis disease estimated by the Kaplan–Meier approach in patients with and without hepatitis C infection. HCV = hepatitis C virus.

In the follow-up time, active TB disease was diagnosed in 36 patients with HCV infection, and the incidence rate was 134.1 (95% confidence interval [CI], 95.3–183.6) per 100,000 person-years. In non-HCV-infected controls, 242 patients had active TB disease, corresponding to an incidence rate of 89.1 (95% CI, 78.4–100.8) (Table 2). The risk of developing active TB disease was significantly higher in HCV-infected patients than in non-HCV-infected patients, with an incidence rate ratio of 1.51 (95% CI, 1.06–2.14; *P* = 0.014). The documented incidence rate of active TB disease was 65 in LHID-2005,^10^ comparable with the data from the Center for Disease Control (CDC) in Taiwan. By Kaplan–Meier approach, the cumulative incidences of active TB disease were higher in HCV-infected patients than controls (*P* = 0.021) (Figure 2).

**TABLE 2:**
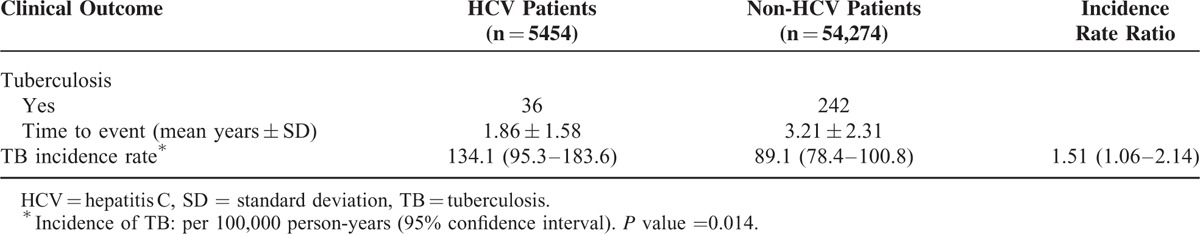
Incidence Rate of Active TB Disease Among HCV and Non-HCV Patients

### Time-Dependent Univariate and Multivariate Analysis for Independent Risk Factors of Active TB Disease

Cox regression analyses with stepwise selection procedures based on the likelihood ratio were performed (Table 3). Crude HR of active TB disease among HCV-infected patients was 3.48 (95% CI, 2.02–6.00). In the multivariate model, HCV infection was independently associated with active TB disease (adjusted HR, 3.20; 95% CI, 1.85–5.53). Older age, male sex, urban residence, alcoholism, and presence of COPD were also independent risk factors for developing active TB disease.

**TABLE 3:**
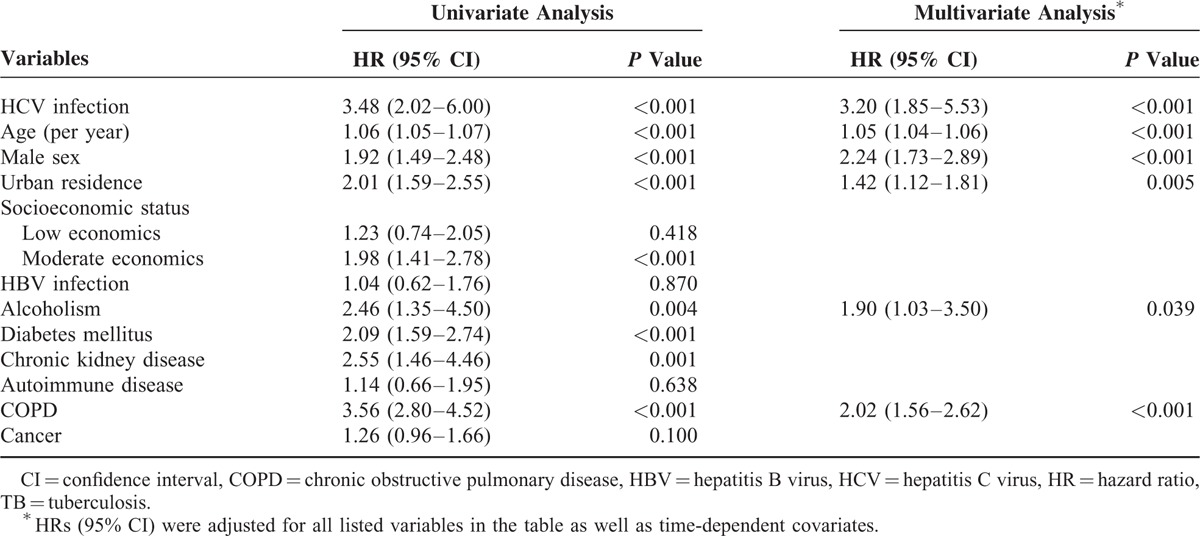
Univariate and Multivariate Adjusted Hazard Ratios of Covariates for Risk Factors Associated With Active TB Disease in the Entire Study Population

The association between HCV infection and active TB disease was robust when we analyzed the data in different multivariate model. Taking competing risk event into consideration, the modified Cox proportional hazards model demonstrated an adjusted HR of 2.11 (95% CI, 1.39–3.20; *P* < 0.001) (Table 4). The adjusted HR was 3.16 (95% CI, 1.82–5.47) after controlling immunosuppressive agents during the observation period.

**TABLE 4:**
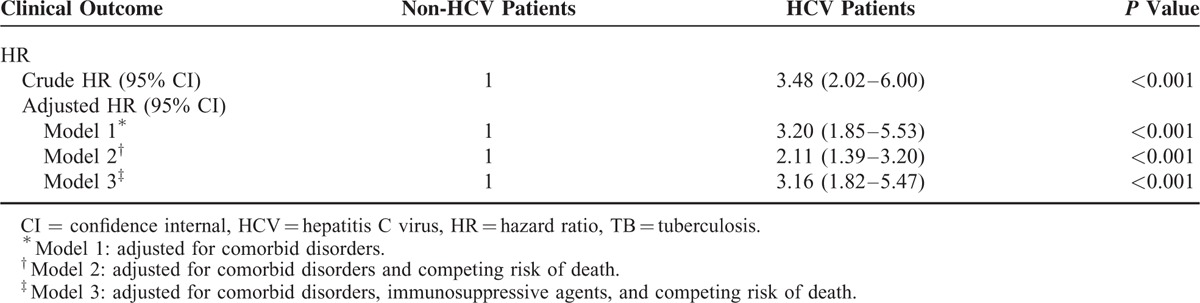
Crude and Adjusted Hazard Ratio of Active TB Disease Among HCV and Non-HCV Patients

### Multivariate Stratified Analyses for Associations Between HCV Infection and Active TB Disease

**FIGURE 3:**
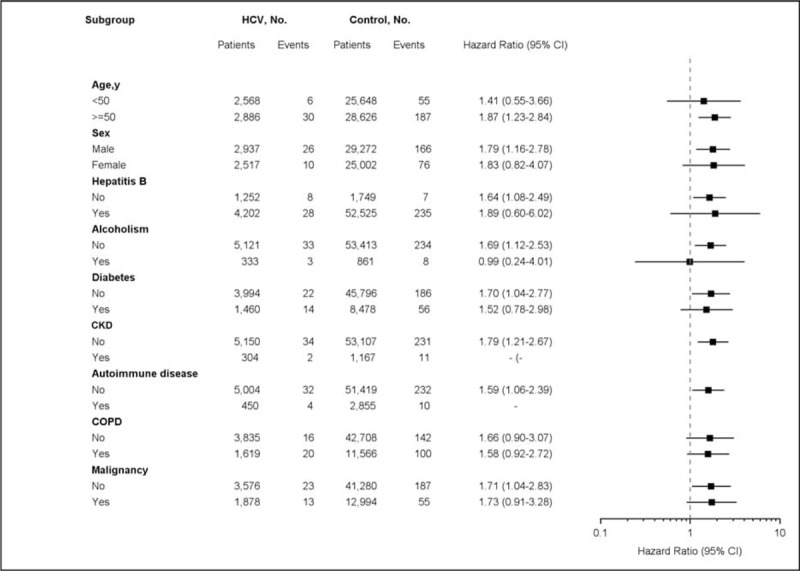
Multivariate stratified analyses for the association of HCV infection and active TB disease. HCV infection was significantly associated with a higher risk of active TB disease in most strata, except for the stratum by age <50 y, HBV infection, alcoholism, and CKD because of the extremely small case numbers of active TB disease. CKD = chronic kidney disease, COPD = chronic obstructive pulmonary disease, HCV = hepatitis C virus, TB = tuberculosis.

The association between HCV infection and active TB disease was further analyzed in Cox regression models stratified by other risk factors associated with TB infection, with adjustment for age, sex, economic status, geographic area, and comorbid disorders. The results showed that HCV infection caused higher risk of active TB disease significantly in most strata, except for the stratum by age <50 years, HBV infection, alcoholism, and CKD (Figure 3).

## DISCUSSION

TB and HCV infections are both common infectious diseases. Although these 2 diseases have similar epidemiological risk factors,^6,7^ and HCV infection is frequently found in patients with TB,^11^ the association between these 2 infections has never been comprehensively studied.^8^ We used the NHIRD of a large-scale cohort and performed multivariate analysis adjusting for possible confounders to investigate the impact of HCV infection on the risk of developing active TB disease. Our results showed that the risk of developing active TB disease was significantly higher in patients with HCV infection than in those without. The association remained robust after accounting for other risk factors of TB, immunosuppressive agents, and even competing risk of death. In addition to HCV infection, our study also found that old age, male sex, urban residence, COPD, and alcoholism were independently associated with active TB disease.

A cross-sectional, hospital-based study showed that TB patients have high prevalence of HCV coinfection but low prevalence of HIV coinfection.^11^ Another hospital-based case-control study during 1992 to 1999 demonstrated HCV-infected patients had a significantly higher prevalence of TB disease. After excluding immunocompromised patients with HIV infection, organ transplant, and liver cirrhosis, HCV remained significantly associated with TB infection (adjusted odds ratio, 1.43; 95% CI, 1.25–1.64).^8^ In Taiwan, coinfection of HIV in HCV-infected patients was rare, because most HCV patients were not drug abusers but infected through medical procedure in hospital at that period of time. Owing to the low incidence and prevalence rate of HIV infection in Taiwan, we excluded patients with HIV infection in case/control group in this present cohort.

HCV infection, rather than HBV infection, has been reported as a risk factor for TB disease after renal transplantation.^12–14^ Our study had similar results that HBV infection was not associated with active TB disease. However, the causal link between HCV infection and TB disease found in this study is biologically plausible. From renal transplantation study, HCV infection has a direct role influencing cellular immune response and favoring a lower rejection rate and an increased rate of opportunistic infections. The HCV-positive kidney transplant recipients present as relative reduction in naïve T cells accompanied by impaired lymphocyte proliferative responses, which is important in the defense against TB.^15^ The deteriorated cellular immune response on account of HCV infection would facilitate the development of intracellular infections. Dendritic cells generated in vitro from peripheral blood of HCV-infected individuals appear impaired in their capacity for antigen presentation,^16^ which correlate with decrease and dysfunction of dendritic cells.^17^ Furthermore, HCV core protein could bind to and inhibit the tumor necrosis factor-alpha (TNF-α) receptor.^18,19^ As TNF-α is a important cytokines for acute TB control,^20,21^ the relation between HCV infection and TB disease could be speculated. HCV infection also has been linked to numerous diseases of immune dysfunction and possible mechanism known as B cell, T cell, natural killer cell, and dendritic cell dysregulation.^22^ In summary, HCV infection may increase the risk of active TB disease by its immunomodulatory effects. Once the relationship of these 2 infections is established by future studies, active TB disease might be considered as one of the extrahepatic complications in HCV infection.

This study has a number of strengths. First, this is a large-scale follow-up study using the well-established nationwide database NHIRD. The study cohort was highly representative for general population. Second, claims for each insured can be tracked across time in the NHIRD. In the present study, all claims of different medical institutes during the study period were obtained for analysis. This can avoid the bias of patient dropout in most longitudinal studies and minimize the possibility of recall bias. Third, we analyzed the data in multivariate models and control many confounding factors and competing death risk, so the results are suggested robust. Nevertheless, we acknowledge several limitations of our study. First, the diagnosis of active TB disease from administrative data is based on diagnostic codes and prescription history. The results of TB cultures and pathological examinations were not available in our database. In addition, some TB-infected patients who did not claim for medications or died within 2 months of treatment were not included. Nonetheless, the incidence of active TB disease in our HCV-infected patients was still higher than that in general population in Taiwan.^2,23^ In Taiwan, active TB disease is an infectious disease of obligatory notification. Doctors are required to report incident cases of TB infection to the Taiwan CDC within 1 week of TB diagnosis or prescription of anti-TB drugs. In the present study, patients were defined as having incident active TB diseases when they had both TB diagnosis and insurance claims for anti-TB drugs for ≥60 days. We believed our definition was convincing. Second, some of the personal information that may contribute to TB diseases was not included in dataset, such as smoking, alcohol consumption, nutritional status, occupational exposure, and contact history of TB. As smoking is associated with pulmonary TB,^24^ it is possible that higher risk of developing active TB disease in male sex found in the present study were at least partially driven by the higher rate of smoking in men. Third, although we excluded TB cases diagnosed before HCV infection, we were unable to differentiate whether the active TB disease was because of endogenous reactivation or recent transmission. Finally, some subjects without obvious clinical symptoms and did not seek medical attention would had been excluded by ICD-9 coding for diagnosis of HCV infection. Therefore, it is possible that some patients with asymptomatic HCV infections were included in the control group. However, if chronic HCV infection is associated causally with active TB disease, this misclassification would likely bias the results toward the null hypothesis and therefore underestimate of the risk. In addition, chronic hepatitis and liver cancer are major public health problems in Taiwan, so the government has developed practical guidelines and screening programs for viral hepatitis. In Taiwan, most HCV patients are asymptomatically diagnosed by extensive screening programs in the community. Thus, the risk of misclassification is minimal.

## CONCLUSION

The present study provides epidemiological evidence that HCV infection is associated with a higher risk of active TB disease. Prolonged fever or chronic respiratory symptoms in HCV-infected patients should raise the suspicion of active TB disease. The deterioration of cellular immune response by HCV infection may explain the association between HCV infection and active TB disease, but further studies are needed to understand the underlying mechanisms.
